# Effect of (Cd:Zn)S Particle Concentration and Photoexcitation on the Electrical and Ferroelectric Properties of (Cd:Zn)S/P(VDF-TrFE) Composite Films

**DOI:** 10.3390/polym9120650

**Published:** 2017-11-28

**Authors:** Sebastian Engel, David Smykalla, Bernd Ploss, Stephan Gräf, Frank A. Müller

**Affiliations:** 1 Otto Schott Institute of Materials Research (OSIM), Friedrich Schiller University Jena, Löbdergraben 32, 07743 Jena, Germany; sebastian.engel@uni-jena.de (S.E.); stephan.graef@uni-jena.de (S.G.); 2 Department of SciTec, University of Applied Sciences Jena, Carl-Zeiss-Promenade 2, 07745 Jena, Germany; david.smykalla@eah-jena.de (D.S.); bernd.ploss@eah-jena.de (B.P.); 3 Jena Center for Soft Matter (JCSM), Friedrich Schiller University Jena, Philosophenweg 7, 07743 Jena, Germany; 4 Center for Energy and Environmental Chemistry (CEEC), Friedrich Schiller University Jena, Philosophenweg 7a, 07743 Jena, Germany

**Keywords:** composite, ferroelectric polymer, semiconductor, photoexcitation

## Abstract

The influence of semiconductor particle concentration and photoexcitation on the electrical and ferroelectric properties of ferroelectric-semiconductor-composites was investigated. For this purpose, 32 µm thin films of poly(vinylidene fluoride-*co*-trifluoroethylene) with (Cd:Zn)S particle concentrations of between 0 and 20 vol % were fabricated and characterized by scanning electron microscopy, Fourier transformed infrared spectroscopy, X-ray diffraction, and optical spectroscopy. It was shown that the particle concentration has only a negligible influence on the molecular structure of the polymer but strongly determines the optical properties of the composite. For (Cd:Zn)S particle concentrations below 20 vol %, the I-V characteristics of the composites is only marginally affected by the particle concentration and the optical excitation of the composite material. On the contrary, a strong influence of both parameters on the ferro- and pyroelectric properties of the composite films was observed. For particle fractions that exhibit ferroelectric hysteresis, an increased remanent polarization and pyroelectric coefficient due to optical excitation was obtained. A theoretical approach that is based on a “three phase model” of the internal structure was developed to explain the observed results.

## 1. Introduction

Due to their low processing temperatures, large electrical resistivity, and high flexibility polymer based ferroelectric materials are of particular interest to engineer flexible electronic devices, such as energy harvesting systems, memory devices, and sensors [1,2,3,4,5,6,7,8,9]. In this context, poly(vinylidene fluoride) (PVDF) is a promising candidate due to its extraordinary ferroelectric properties and the feasibility of thin film processing. PVDF is a semi-crystalline polymer that exhibits at least four polymorphs (α-, β-, γ-, δ-) [10]. Under normal conditions, it crystalizes from melt to the non-polar α-phase [10]. The polar and ferroelectric β-phase can be fabricated by mechanical stretching of the α-phase [11]. An alternative material is given by the copolymer poly(vinylidene fluoride-*co*-trifluoroethylene) (P(VDF-TrFE)), which exhibits a structure that is well ordered, polar, and analogous to that of the β-phase of PVDF [12,13]. In line with literature, this ferroelectric phase of P(VDF-TrFE) is also referred to as β-phase in this study. P(VDF-TrFE) can directly crystallizes into this ferroelectric β-phase, independent of processing routes or post-treatment procedures, if a content in the range of 20–45 mol % TrFE is adjusted [14]. In addition, the solubility of P(VDF-TrFE) in various solvents makes the material attractive for flexible electronics fabricated by spin coating [15], dip coating [16], and screen printing [17]. Beyond, the suitability to disperse diverse (nano-)particles into the polymer allows for it to tailor the electrical and ferroelectric materials properties. Graz et al. demonstrated that a polymer-ceramic nanocomposite consisting of ferroelectric ceramic particles and P(VDF-TrFE) is able to detect either pressure or temperature [18,19]. Furthermore, it was shown that the addition of ceramic or metallic nanoparticles can enhance the ferroelectric properties of P(VDF-TrFE), resulting in an increased remanent polarization or piezoelectric coefficient [20,21,22,23,24,25]. Poling of the inclusions, as well as the matrix material of a ferroelectric composite with 0–3 connectivity usually requires a two-step poling process with a change of the matrix conductivity between the two steps [19]. This change of the matrix conductivity has been realized by temperature variation. Furthermore, for an optimized coupling of the pyroelectric or piezoelectric activity from the inclusions of a ferroelectric 0–3 composite to the electrodes the matrix material must have well specified conductivity [26,27]. This optimum conductivity depends on the operation frequency of the pyroelectric or piezoelectric sensor and has been realized by doping of the matrix material [28]. PVDF and P(VDF-TrFE) materials are partially crystalline. Here, the crystallites are embedded in the matrix of less ordered or amorphous material, i.e., physically these polymers can be considered as 0–3 composites, and the considerations regarding charge transport in the matrix discussed for chemically heterogeneous 0–3 composites also apply to these polymers. For the poling process, as well as for the sensor operation, it would be highly beneficial if the conductivity of the matrix could be controlled by an external parameter other than temperature. Therefore, we suggest to introduce photoconduction by adding photoconductive particles and effectively forming a three phase composite of photoconducting particles into the partially crystalline ferroelectric matrix. Photoconduction will allow a wider variation of conductivity than temperature variation, i.e., the poling process will become faster and more efficient. Furthermore, the easy variation of conductivity by illumination allows for a variation of conductivity of a sensor element in operation and therefore an optimization to the current operation frequency. Thus, one sensor element will become applicable to a wide range of operation frequencies. Recently, composite membranes of P(VDF-TrFE) and semiconductor particles, such as titanium dioxide, have attracted attention concerning their photocatalytic performance [29,30]. In our study, we focus on investigating the ferroelectric properties of thin composite films that consist of (Cd:Zn)S particles dispersed in P(VDF-TrFE). In addition to the evaluation of the influence of the (Cd:Zn)S particle concentration, the influence of an optical excitation on the electrical and ferroelectric properties of the composite is investigated. (Cd:Zn)S particles were used due to their bandgap, which allows optical excitation of the particles situated within the P(VDF-TrFE) matrix. In addition, they exhibit a relatively small size and they are commercially available.

## 2. Materials and Methods

### 2.1. Sample Preparation

Composite samples were prepared by dispersing 100 mg 70/30 Poly(vinylidene fluoride trifluoroethylene) [P(VDF-TrFE)] (Piezotech Arkema, Pierre-Benite Cedex, France) and (Cd:Zn)S powder (<500 nm, Kremer Pigmente, Aichstetten, Germany) in a 100 mL methyl ethyl ketone (Carl Roth, Karlsruhe, Germany) ultrasonic bath, followed by magnetic stirring at 50 °C for 180 min. Specific concentrations between 0 and 20 vol % (Cd:Zn)S were realized by varying the amount of the (Cd:Zn)S powder between 0 and 50 mg. They were calculated using the respective densities of P(VDF-TrFE) (ρ = 1.8 g/cm³) and (Cd:Zn)S (ρ = 4.5 g/cm³). Composite foils with a final thickness of 32 µm were fabricated by evaporation of the methyl ethyl ketone from the prepared solution in a petri dish and subsequent compression molding for 3 min at 170 °C and 30 kN. Compression molding enables more homogenous composite samples concerning thickness, surface quality, and (Cd:Zn)S particle distribution. Finally, circular electrodes were deposited by sputtering gold on the top surface (thickness: 30 nm, diameter: 6 mm) and on the bottom surface (thickness: 60 nm, diameter: 10 mm), respectively, to enable electrical contacting and in order to allow for an optical excitation from the top surface of the foils.

### 2.2. Characterization

The phase composition and structure of the composite films was analysed by attenuated total reflection Fourier transformed infrared spectroscopy (ATR-FTIR) (Alpha-P, Bruker, Billerica, MA, USA), with a step size of 1.4 cm^−1^ in the range of 700 to 1400 cm^−1^ using a diamond crystal. The measured spectra were normalized to the most intense peak of the ferroelectric β-phase of P(VDF-TrFE) at 878 cm^−1^ [31]. The crystalline phase composition was characterized by X-ray diffraction (XRD) (D5000, Siemens Diffractometer, München, Germany) using CuK_α_-radiation (λ = 0.15405 nm) at an operating voltage of 40 kV and an operating current of 30 mA. The scanning rate was 0.02°/s for the 2θ range of 10°–60°. The optical properties were characterized by UV-Vis transmission spectroscopy of the composite films without the gold electrodes. For this purpose, a 2 inch integrating sphere (IS236A-4, Thorlabs, Newton, NJ, USA) was used to exclude scattering effects resulting from the (Cd:Zn)S particles and the polymer. A 75 W xenon arc lamp (Tunable Power Arc Illuminator, OBB, Edison, NJ, USA) was used as the radiation source, emitting a spectrum between 400 and 1050 nm. The radiation was guided through an optical fiber onto a telescope that served as a collimator. The diffused light transmitted through the sample was uniformed by multiple scattering reflections at the sphere walls and detected by an optical spectrometer (Maya2000 Pro, Ocean Optics, Ostfildern, Germany).

### 2.3. Electrical, Ferroelectric and Pyroelectric Properties

**Figure 1:**
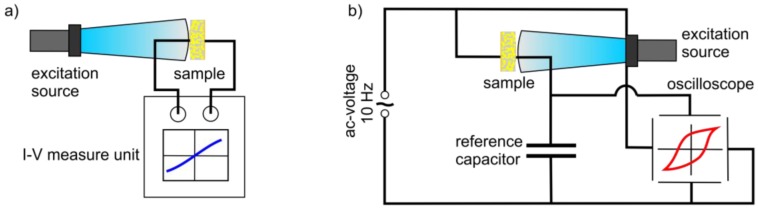
Experimental setup to analyse the electrical and ferroelectric properties of the poly(vinylidene fluoride-*co*-trifluoroethylene) (P(VDF-TrFE)) composites in dependence on (Cd:Zn)S particle concentration and photoexcitation using (**a**) an I-V measurement setup and (**b**) a Sawyer-Tower circuit.

The electrical and ferroelectric properties of the composite samples were investigated by performing I-V and polarization measurements, using the experimental setup shown in Figure 1. The I-V characteristic was characterised by a precision source/measure unit (B2901A, Keysight Technologies, Santa Rosa, CA, USA) applying a “double linear” voltage regime between −200 V and 200 V (Figure 1a). For the composite with the largest (Cd:Zn)S concentration of 20 vol %, the voltage was set to ±50 V in order to prevent from electrical breakdown during the measurement. The ferroelectric hysteresis loops of the composite foils were recorded utilizing a Sawyer-Tower circuit at a frequency of 10 Hz and a voltage loop with a peak-to-peak value of 3.2 kV (Figure 1b). The capacity of the reference capacitor was 1 µF. Both characterisation methods were conducted with and without optical excitation in order to investigate the influence of an optical excitation on the electrical and ferroelectric properties of the composite samples. For this purpose, the collimated radiation of a LED with a central wavelength of 460 nm (bandwidth FWHM: 24 nm) and an intensity of 0.2 mW/mm^2^ was used for excitation.

An ac method was used to measure the pyroelectric coefficient. The sample temperature was sinusoidally modulated at a frequency of 10 mHz and a peak amplitude of 1 K using a Peltier element that was regulated by a proportional-integral-derivative (PID) controller [19]. The pyroelectric current signal was amplified with a current-to-voltage converter and measured with a lock-in amplifier.

## 3. Results

### 3.1. Microstructure and Optical Properties

**Figure 2:**
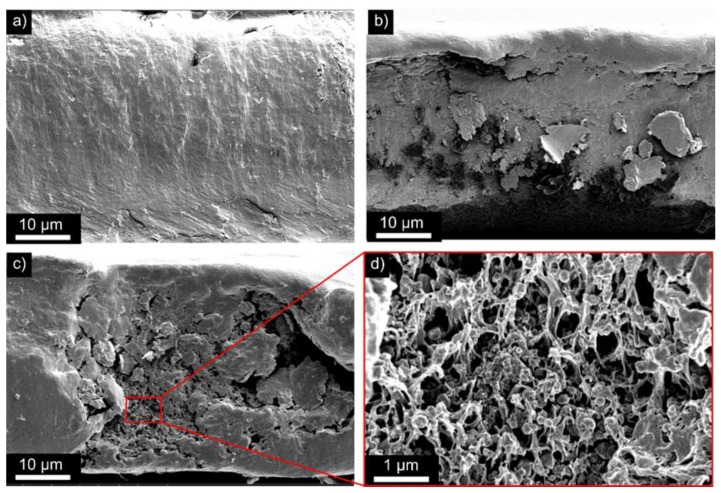
SEM micrographs of cross-sections of the P(VDF-TrFE) composite material with a (Cd:Zn)S particle concentration of (**a**) 1 vol %, (**b**) 10 vol %, and (**c**,**d**) 20 vol %.

Figure 2 shows scanning electron microscopy (SEM) micrographs of cross-sections of the P(VDF-TrFE) composite material, with a (Cd:Zn)S particle concentration of 1 vol % (Figure 2a), 10 vol % (Figure 2b), and 20 vol % (Figure 2c). It becomes evident, that the surface of the cross-section of the 1 vol % composite is very homogenous and uniform. A similar morphology can be observed for the sample with a concentration of 10 vol %, which is characterized by an increased roughness due to the appearance of agglomerates of particles. The formation of agglomerates becomes more pronounced with an increasing (Cd:Zn)S particle concentration, as revealed by the 20 vol % sample (Figure 2d). Here, the agglomerates form penetrating networks covering the complete thickness of the composite film (Figure 2c).

**Figure 3:**
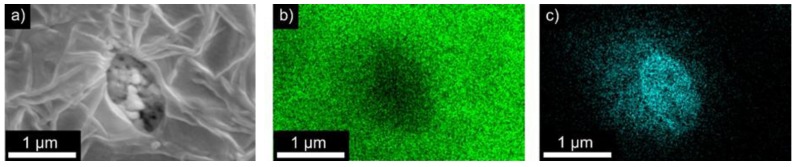
SEM/EDX analysis of a P(VDF-TrFE) composite material with a (Cd:Zn)S particle concentration of 10 vol %: (**a**) SEM micrograph; (**b**,**c**) corresponding EDX maps for fluorine and sulfur, respectively.

The occurrence of (Cd:Zn)S particles in the composite material is also confirmed by energy-dispersive X-ray (EDX) analyses. Figure 3a shows the SEM micrograph of the composite material with a (Cd:Zn)S particle concentration of 10 vol %. The corresponding EDX maps indicate the content of fluorine as an element related to the polymer matrix (Figure 3b), and of sulfur as an element that is related to the (Cd:Zn)S particles (Figure 3c). It becomes evident, that specific areas in the SEM micrograph can be allocated to sulfur enriched regions, i.e., to (Cd:Zn)S particles.

**Figure 4:**
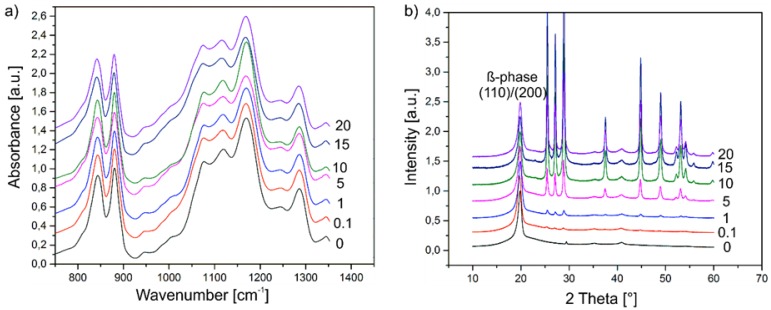
(**a**) Attenuated total reflection Fourier transformed infrared spectroscopy (ATR-FTIR) spectra and (**b**) X-ray diffraction (XRD) spectra of P(VDF-TrFE) composite films with different (Cd:Zn)S particle concentration (0 = 0 vol %, 0.1 = 0.1 vol %, 1 = 1 vol %, 5 = 5 vol %, 10 = 10 vol %, 15 = 15 vol %, 20 = 20 vol %).

ATR-FTIR spectra of the composite films with a (Cd:Zn)S particle concentration between 0 and 20 vol % are illustrated in Figure 4a. All spectra are very similar showing main peaks at 840, 878, and 1167 cm^−1^. They indicate the presence of the β-phase of P(VDF-TrFE) [31]. The similar behaviour of all the samples indicates a negligible effect of the added (Cd:Zn)S particles on the molecular structure of the films, and therefore on the internal structure of the P(VDF-TrFE) matrix [21,32]. The corresponding XRD spectra exhibit several characteristic peaks that confirm the presence of the polar β-phase (Figure 4b). This includes the strong diffraction peak at 2θ = 19.9° ((110) and (200) orientation plane), as well as two smaller peaks at 2θ = 35.5° (001) and 2θ = 40.9° (201,111) [33]. With increasing particle concentration, the characteristic peaks of crystalline (Cd:Zn)S appear in the spectra [34]. SEM, EDX, ATR-FTIR, and XRD measurements indicate that the (Cd:Zn)S particles are present as an additional, separate phase and that they have only a negligible influence on the internal crystalline and amorphous (semi-crystalline) structure of the P(VDF-TrFE) matrix.

**Figure 5:**
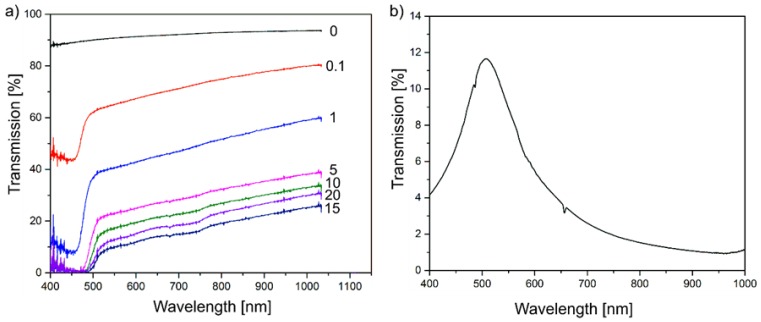
UV-Vis Transmission spectra of (**a**) (Cd:Zn)S/P(VDF-TrFE) composite films (0 = 0 vol %, 0.1 = 0.1 vol %, 1 = 1 vol %, 5 = 5 vol %, 10 = 10 vol %, 15 = 15 vol %, 20 = 20 vol %) without gold electrodes and (**b**) the single gold electrode with a thickness of 30 nm.

Figure 5a shows UV-Vis transmission spectra of P(VDF-TrFE) composite films, with varying (Cd:Zn)S particle concentration without gold electrodes in dependence on the illumination wavelength. The pristine P(VDF-TrFE) polymer exhibits a transmission of about 90%, which is almost constant in the investigated wavelength range. Deviations from a total transmission are related to reflections at both composite surfaces and to scattering effects. The results indicate a remarkable decrease of the transmission in the entire wavelength range, with an increasing concentration of (Cd:Zn)S particles. In the wavelength range above 490 nm, this can be explained by an increasing scattering. As an exception, the composite with 20 vol % (Cd:Zn)S exhibits a slightly larger transmission for this wavelengths range when compared to the 15 vol % (Cd:Zn)S composite film. This behaviour might be explained by the observed agglomeration effects of the particles (Figure 2c,d), which result in a reduced number of scattering centres, and therefore in a reduction of scattering effects. As a consequence, the transmission of light with λ ≥ 490 nm increases. It becomes evident, that all spectra of P(VDF-TrFE) composite films including (Cd:Zn)S particles are characterised by a sharp decrease of the transmission at wavelengths below λ = 490 nm, related to the band gap of the (Cd:Zn)S particles. Consequently, irradiation of light with λ ≤ 490 nm leads to an optical excitation of the semiconductor particles, resulting in free charge carriers in the conduction band [35]. In this wavelength range, a volume fraction of at least 5% leads to a vanishingly small transmission, i.e., a maximum absorption. For completeness, the UV-Vis transmission spectra of the gold electrode, with a thickness of 30 nm (Figure 5b) that reveals a transmission peak with a height of about 8% at λ = 460 nm. 

### 3.2. Electrical and Ferroelectric Properties

**Figure 6:**
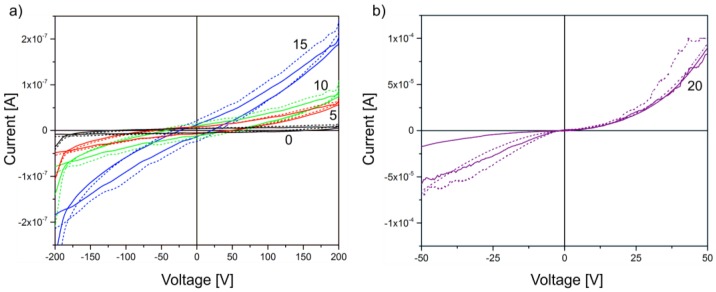
I-V curves of (Cd:Zn)S/P(VDF-TrFE) composite films without (solid lines) and with (dotted lines) optical excitation in dependence on the (Cd:Zn)S particle concentration: (**a**) 0 to 15 vol % (0 = 0 vol %, 5 = 5 vol %, 10 = 10 vol %, 15 = 15 vol %) and (**b**) 20 vol %. The I-V curves for a particle concentration of 0.1 and 1 vol % (not shown in this figure) are very similar to pristine P(VDF-TrFE).

The I-V characteristics of the samples indicate very similar electrical properties of the pristine P(VDF-TrFE) polymer and the P(VDF-TrFE) composites with a (Cd:Zn)S particle concentration of 0.1 and 1 vol %, respectively. As illustrated in Figure 6, a negligible current was detected in the investigated voltage range without and with optical excitation. On the contrary, a particle concentration between 5 and 15 vol % results in a moderate increase of the current flow with an increasing voltage. Nevertheless, a photocurrent that is caused by optical excitation of additional free charge carriers in the (Cd:Zn)S particles of the P(VDF-TrFE) composites can only be detected for 10 and 15 vol %. Consequently, the influence of the optical excitation on the current flow can be assumed to be relatively small. 

**Figure 7:**
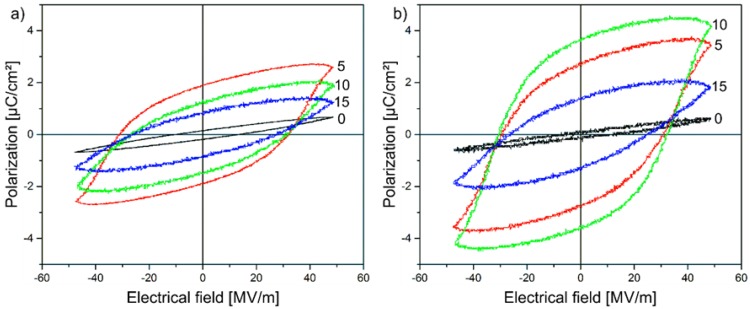
Hysteresis loops of the polarization of P(VDF-TrFE) composite films with different (Cd:Zn)S particle concentrations (0 = 0 vol %, 5 = 5 vol %, 10 = 10 vol %, 15 = 15 vol %) in dependence on the electric field E (**a**) under non-excitation and (**b**) under optical excitation conditions.

The high resistivity determined for all of these samples can be explained by the localisation of the free charge carriers in the (Cd:Zn)S particles and their limited transition into surrounding particles and the polymer matrix, respectively. A remarkable increase of the current flow can be detected for 20 vol % (Cd:Zn)S (Figure 6b). Here, the corresponding current density is three orders of magnitude higher when compared to the samples with a lower (Cd:Zn)S particle concentration. This also concerns the photocurrent that is induced by the optical excitation of the composite materials. The sharply decreased resistivity that is measured for this composite can only be explained by the formation of (Cd:Zn)S agglomerates (Figure 2c,d). They lead to conduction paths that cause an increasing leakage current. Consequently, the current flow at the applied voltage induces a local electrical breakdown, which results in destruction of the sample during the polarization measurement thus making a polarization of the sample impossible. Therefore, concentrations of more than 20 vol % (Cd:Zn)S were not used in the present study. Concerning the other P(VDF-TrFE) composite films with (Cd:Zn)S particle concentration of 0, 5, 10, and 15 vol %, the hysteresis loops of the polarization *P* in dependence on the electric field E with and without optical excitation are shown in Figure 7. The hysteresis curves are displayed as measured, i.e., leakage current has not been subtracted. It has to be noted, that the hysteresis loops of the composite films with the lowest particle concentrations of 0.1 and 1 vol % (not shown in Figure 7) are similar to the pristine polymer. For all of these samples, the maximum utilized electrical field of 50 MV/m was insufficient to obtain a polarization of the material, i.e., only a very small hysteresis loop was detected. Beyond, the photoexcitation of the samples did not influence their ferroelectric behaviour. The measured P-E loops exhibit an optimum particle concentration of about 5 vol % in the non-excited regime, and 10 vol % in the excited regime, respectively, which is required to achieve a pronounced hysteresis loop.

**Figure 8:**
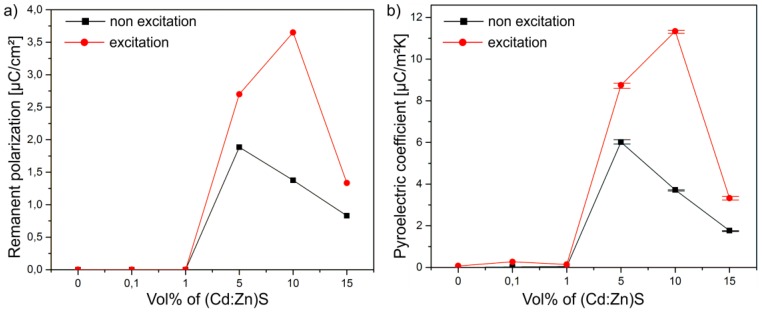
(**a**) Remanent polarization and (**b**) Pyroelectric coefficient of P(VDF-TrFE) composites as a function of the (Cd:Zn)S particle concentration polarized with and without photoexcitation.

The remanent polarization *P*_r_ of the composites (intercept of the loops with the *y*-axis in Figure 7) in dependence on the particle concentration is shown in Figure 8a. It becomes evident that the maximum value of *P*_r_ without optical excitation can be observed for a (Cd:Zn)S particle concentration of 5 vol %. The increase of the concentration up to 15 vol % leads to a decrease of the remanent polarization. However, this influence of the particle concentration is not the only decisive fact. In contrast to the I-V characteristic (Figure 6a), the optical excitation of the samples with a (Cd:Zn)S particle concentration of 5, 10, and 15 vol % has a significant influence on their ferroelectric behaviour (Figure 7b). For all three samples, the hysteresis loops are more pronounced for the excited material, which illustrates the strong influence of the photoexcitation on the ferroelectric properties. Under photoexcited conditions, the most pronounced hysteresis can be observed for a (Cd:Zn)S particle concentration of 10 vol %. In this case, the optical excitation leads to an increase of *P*_r_ by a factor of about 2.7 from 1.37 µC/cm^2^ without excitation to 3.65 µC/cm^2^ for the excited material.

These effects are confirmed by pyroelectric measurements of the composite samples. Figure 8b shows the pyroelectric coefficient *p* of the P(VDF-TrFE) as a function of the (Cd:Zn)S particle concentration of the samples that are polarized with and without optical excitation. Obviously, the pyroelectric coefficient p exhibits a dependency on the particle concentration similar to the remanent polarization *P*_r_ illustrated in Figure 8a. Analogue to *P*_r_, the maximum value of the pyroelectric coefficient for the non-excited composite can be observed at 5 vol % (*p* = 6 µC/m^2^K) and for the excited composite at 10 vol % (*p* = 11.4 µC/m^2^K), respectively. 

## 4. Discussion

In the present study, the electrical and ferroelectric properties of P(VDF-TrFE) composite films with dispersed (Cd:Zn)S particles have been investigated with respect to the influence of the (Cd:Zn)S particle concentration and the photoexcitation of the composite material. It was shown that the electrical conductivity kept was almost unaffected by the dispersion of different particle concentrations, except for the composite with a (Cd:Zn)S particle concentration of 20 vol %. Contrary to composites with small particle concentrations (0, 0.1, and 1 vol %), larger concentrations of 5, 10, and 15 vol % showed ferroelectric hysteresis loops that were caused by the utilization of an electrical field of 50 MV/m. These hysteresis loops become more pronounced under photoexcitation. 

**Figure 9:**
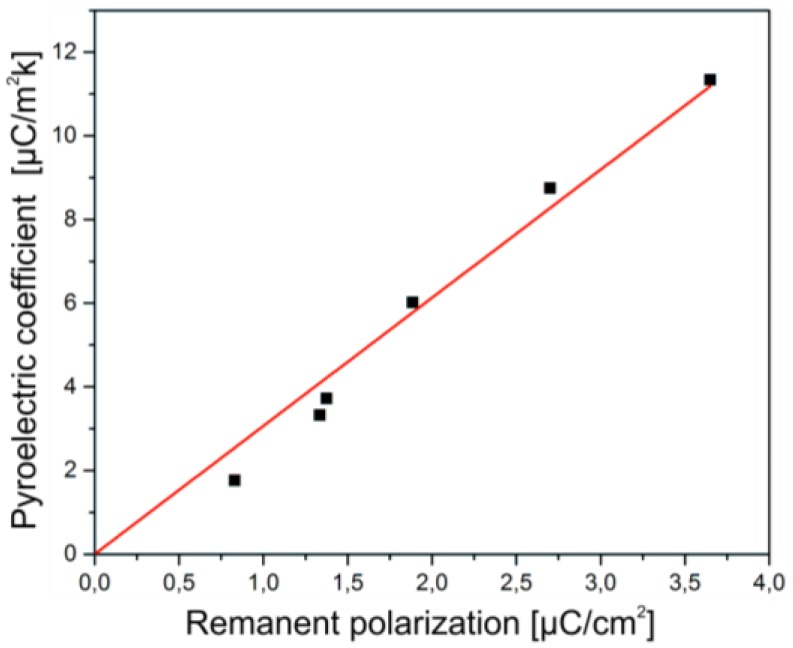
Pyroelectric coefficient of the ferroelectric (Cd:Zn)S/P(VDF-TrFE) composites as a function of the remanent polarization.

The same relationship of the remanent polarization (Figure 8a) and the pyroelectric coefficient (Figure 8b) with respect to the (Cd:Zn)S particle concentration in the excited and the non-excited regime results in a linear correlation between the two coefficients (Figure 9). Both the pyroelectric coefficient as a characteristic property of the pure ferroelectric behavior of P(VDF-TrFE) [36], and the linear correlation between the remanent polarization and the pyroelectric coefficient confirm the negligibly small influence of the leakage current on the shape of the hysteresis loops, as measured with and without optical excitation for the samples containing 10, 15, and 20 vol % (Cd:Zn)S particles. In addition to that, the very low I-V characteristic unveils the displacement current as the main current influencing the P-E hysteresis loops.

**Figure 10:**
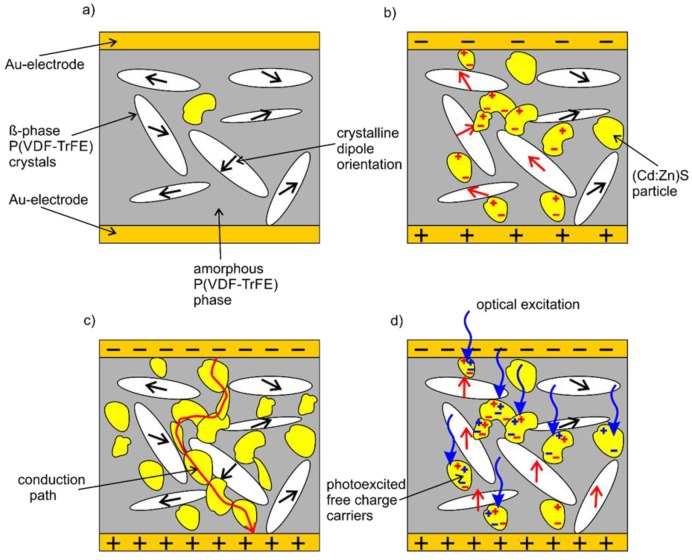
Schematic illustration of the proposed “three phase model”: (**a**) composite film at a (Cd:Zn)S concentration of 0, 0.1 and 1 vol % without ferroelectric behaviour at an electrical field of 50 MV/m, (**b**) dipole orientation of the ferroelectric β-phase of P(VDF-TrFE) at a (Cd:Zn)S concentration of 5, 10 and 15 vol % resulting from surface charge balancing and an increasing effective adjacent electric field, (**c**) the formation of conduction paths at a (Cd:Zn)S particle concentration of 20 vol %, and (**d**) photoexcitation of additional free charge carriers resulting in an enhanced ferroelectric behaviour at (Cd:Zn)S concentrations of 5, 10 and 15 vol %.

The results obtained for the P-E hysteresis loops, the remanent polarization, and the pyroelectric coefficient measured in dependence on the (Cd:Zn)S particle concentration and photoexcitation can be explained by a “three phase model”. In the case of the pristine P(VDF-TrFE), the micro- and nanoscale structure consists of two coexisting phases that are related to the amorphous and the crystalline regions of the polymer (Figure 10a). The amorphous regions are non-ferroelectric and the regions of the crystalline β-phase are ferroelectric due to their dipole moment. It is generally accepted that the pyroelectricity of P(VDF-TrFE) originates in poling-induced dipole orientation in the ferroelectric crystals [36]. In order to achieve a permanent dipole orientation without an external field, the remanent polarization of the crystals has to be compensated by surface and space charges [37]. As illustrated in Figure 10a, the additional dispersion of (Cd:Zn)S semiconductor particles introduces a third phase. The occurrence of this additional phase and the possibility of the photoexcitation of the semiconductor material have to be considered in order to explain the observed properties of the composites. Concerning the composites with low (Cd:Zn)S particle concentrations of 0, 0.1 and 1 vol % (Cd:Zn)S, the maximum utilized electrical field of 50 MV/m was insufficient to realize a polarization of the composite material (Figure 8a). In this case, only a few dipoles are aligned along the direction of the electrical field. However, at a certain concentration, which was found to be around 5 vol %, the following aspects have to be considered. Firstly, the (Cd:Zn)S particles in the amorphous phase of P(VDF-TrFE) are situated very close to each other. Consequently, several crystals and the dipoles of the ferroelectric β-phase of P(VDF-TrFE) are more or less electrically contacted (Figure 10b), and therefore, an essential surface charge balancing of the crystals during the poling-induced dipole orientation can occur. Secondly, the effective adjacent electric field at the crystal β-phase of P(VDF-TrFE) increases due to the decreasing local resistivity of the surrounding phases that is caused by the semiconductor particles dispersed in the amorphous (semi-crystalline) region of P(VDF-TrFE). The combination of both the effects results in a polarizability of the composites at a minimum particle concentration (Figure 10b). The decrease of the polarizability with further increasing (Cd:Zn)S particle concentration can be explained by the combination of a decreasing volume fraction of the polymer crystals and an increasing leakage current, which reduces the effective adjacent electric field at the P(VDF-TrFE) crystal β-phase. This leakage current becomes dominant at a certain (Cd:Zn)S particle concentration, which was found to be about 20 vol %, due to the formation of conduction paths by the particles (Figure 10c). Finally, the enhancement of the ferroelectric properties due to the photoexcitation of the semiconductor particles results from the excitation of free charge carriers. These additional free charges cause an increase of two effects: surface charge balancing and the increase of the effective adjacent electric field at the P(VDF-TrFE) crystal β-phase (Figure 10d).

## 5. Conclusions

Electrical and ferroelectric properties of (Cd:Zn)S/P(VDF-TrFE) composite films have been investigated concerning the influence of the (Cd:Zn)S particle concentration and the photoexcitation of the composite material. It was shown that both of the parameters provide alternative tools to adjust the ferroelectric properties of ferroelectric-semiconductor-composites. Based on the systematic discussion of the experimental results using a proposed “three phase model”, our findings facilitate to realize new applications in the fields of (optical) sensors, memory devices, and smart materials by combining ferroelectrics and photoexcitation.
